# DNA–CTMA Matrix Influence on Rhodamine 610 Light Emission in Thin Films

**DOI:** 10.3390/polym15143105

**Published:** 2023-07-21

**Authors:** Adrian Petris, Petronela Gheorghe, Ileana Rău

**Affiliations:** 1 National Institute for Laser, Plasma and Radiation Physics, 409 Atomistilor Street, 077125 Magurele, Romania; 2 Faculty of Chemical Engineering and Biotechnologies, University POLITEHNICA Bucharest, 1-7 Polizu Street, 011061 Bucharest, Romania

**Keywords:** biopolymers, biocomposites, photobleaching, DNA, Rhodamine 610

## Abstract

Due to the increased application of lasers in different fields (industry, medicine, etc.), there is a growing need for new laser sources with good beam quality and variable emission wavelength. At the same time, for environmental reasons, the obtaining of novel eco-friendly active optical materials, such as those based on the deoxyribonucleic acid (DNA) biopolymer, with optimal light emission properties, is of high interest. The results obtained in this study of the temporal dependence of the transmittance and of the light emission in thin films of DNA–CTMA–Rhodamine 610 (at different Rhodamine concentrations) (DNA–CTMA–Rh610), when they are illuminated with continuous wave laser light at 532 nm (frequently used in the optical pumping of dye lasers), are presented and discussed. The transmittance results obtained for thin film samples are compared to those obtained for the DNA–CTMA–Rh610 solutions in butanol, from which the films have been made, and also with those obtained for Rh610 solutions in butanol with the same concentrations. The investigation was performed in order to assess the influence of the DNA-CTMA and of the green laser light at 532 nm wavelength on relevant chromophore properties such as light transmission and fluorescence emission. The results obtained revealed that the DNA–CTMA matrix has an active influence on the Rhodamine 610 emission, in the whole range of concentrations of the investigated samples.

## 1. Introduction

The environmental protection issues made the replacing of synthetic polymers with biopolymers a “must”. One of the biopolymers investigated in this respect is deoxyribonucleic acid (DNA) due to several advantages of its use, briefly mentioned below. It is a “green” material, with a fast degradation time in nonprotected environments and originates from the waste produced by the food processing industry. Therefore, DNA could be obtained from renewable resources, and it can be considered a low-cost material.

In 2007, A. Steckl asserted in the first number of Nature Photonics that “DNA—the molecule of life—is an exciting new material for fabricating photonic devices with enhanced properties” [1]. At that moment, his statement was based mainly on the BioLEDs proposed by J. Grote laboratory. Also, G. Overton considered in 2008 that the next-generation optoelectronics leverage DNA biopolymers [2]. Since then, much research has been performed in order to propose and to develop new DNA-based materials with different applications in photonics [3]. Despite the fact that these DNA-based materials have been continuously studied for more than 15 years, they are still intriguing materials. DNA biopolymer photonics represents an innovative platform toward sustainable technology [4]. Recently, K. Dunn and A. Elfick [5] demonstrated that DNA molecules, with their ability to be functionalized, allow specific nanostructures to be obtained. In this way, the doors of DNA nanotechnology for the construction of many novel devices and systems are opened.

DNA-based materials exhibit low light propagation losses and interesting electrical properties. With certain surfactants as, e.g., cetyltrimethylammonium chloride (CTMA), DNA forms water-insoluble complexes (DNA–CTMA), which, on the other hand, are soluble in a large number of solvents (e.g., ethanol, butanol, and isopropanol), thus facilitating its functionalization with different chromophores [6]. Important advantages of using DNA biopolymer as a host for chromophores comprise the increased stability of embedded dyes, numerous possibilities for rendering it functional, versatility, and excellent thin film processability. The functionalization of DNA with different chromophores allows the tuning of the optical properties of the resulting DNA complexes for targeted photonic functionalities and applications, e.g., frequency conversion (third-harmonic generation) [7,8,9], light-induced spatial phase modulation [10,11,12], light-induced holographic diffraction gratings [13], and laser emission [14]. A review of the recent results obtained on DNA-based optoelectronics and photonics, along with some challenges and perspectives of DNA applications in these fields, is presented in [15].

The specific double-stranded helical structure of DNA molecules provides a large free volume for doping molecules and consequently can be used to obtain sufficiently fast photo-induced responses and improved nonlinear optical properties in dye–DNA compounds [11,16,17,18,19]. In addition, it was demonstrated that DNA—Rhodamine 610 films show an amplified spontaneous emission (ASE) threshold and gain a coefficient similar to that obtained when the chromophore was embedded in synthetic polymers [20].

When a laser beam interacts with an optical material, the absorbed light locally increases the temperature of the material. The light-induced temperature rise in the material is inversely proportional to its thermal conductivity. DNA and DNA–CTMA have thermal conductivity values seven times and five times, respectively, larger than, e.g., that of the synthetic polymer PMMA (polymethylmethacrylate), widely used as optical material [21]. Therefore, due to the high thermal conductivity of DNA and DNA–CTMA, their laser heating, as well as that of materials that use the DNA biopolymer as a matrix, is lower compared to other optical materials with similar absorption of the laser light. This is an advantage of using DNA-based materials in photonics.

As matrices for luminophores, the DNA–surfactant complexes appear to be very promising for solid-state laser fabrication. Several laboratories have reported the enhancement of the photoluminescence quantum yield of certain luminophores in DNA and DNA–CTMA matrices, as well as the increase in the concentration quenching limit [22]. This favorable influence of the DNA environment on light emission performance is also seen in the low threshold for amplified spontaneous emission (ASE) [23,24,25,26,27,28].

To be effective in applications based on light emission and amplification, the optically active material must efficiently absorb the optical pumping light and must have efficient photoluminescence. The temporal evolution of these two properties of the active material under illumination with pumping light is important in the investigation of the photochemical stability of the active material, which is a complex process.

In [13], it has been shown that the transmittance of an Ar laser beam (514.5 nm wavelength) through a DNA–CTMA–Rh610 film increases slower than through a PMMA–Rh610 film during their illumination at the mentioned wavelength. The increase in transmittance was attributed to the photo-degradation process of Rh610 dye in the two considered matrices. This process, slower in DNA–CTMA–Rh610 than in PMMA–Rh610, indicates that the protection of Rh610 molecules against photo-degradation is better in the DNA–CTMA matrix than in PMMA one. The superior photo-chemical stability is important for photonic applications of compounds containing the Rh610 dye.

In this paper, the well-known luminophore Rhodamine 610 (Rh610) was used in studying, for the first time, to the best of our knowledge, the influence of the continuous wave (c.w.) green laser light at a 532 nm wavelength on the transmittance of DNA–CTMA–Rh610 thin films at this wavelength, compared to the transmittance of DNA–CTMA–Rh610 solutions in butanol from which they have been made and also to the transmittance of Rh610 solutions in butanol with the same concentrations. The temporal evolution of the fluorescence light emitted by the Rh610 dye in the thin films of DNA–CTMA–Rh610, excited with c.w. laser light at a 532 nm wavelength (frequently used as pump light in dye lasers), is also investigated. The DNA biopolymer is functionalized with CTMA surfactant to make it soluble in the same solvent (butanol) as Rh610. The influence of the green laser light at a 532 nm wavelength and of the DNA–CTMA matrix on the absorption of the films and on the Rh610 light emission is discussed. The obtained results suggest that these materials can be used for applications based on light emission, such as dye-doped solid-state lasers.

## 2. Materials and Methods

DNA from Ogata Research Laboratory, Ltd., Chitose, Japan and Rhodamine 610, from Exciton company, Dayton, USA were used to prepare the solutions of DNA–CTMA–Rhodamine 610 in butanol (30 g/L DNA–CTMA). The thin films were prepared via the spin-coating technique (WS—400B—6NPP/LITE spin coater, Laurell Technologies Corporation, North Wales, PA, USA) using very well cleaned microscope glass slides as support. The glass slides were cleaned according to a procedure developed in the laboratory, based on the personnel’s experience, a procedure which assures that inorganic as well as organic traces are removed. Basically, it consists of rinsing the glass slides with NaOH solution and alcohol. During the cleaning process, the slides were ultrasonicated and heated to 400 °C. Throughout the procedure, the glass plates were handled with tweezers, and, after cleaning, they were kept in closed boxes in order to avoid their contamination.

The thicknesses of the thin films deposited via spin-coating were measured through profilometry using the DEKTAK 120 profilometer model (KLA Tencor, Milpitas, CA, USA). The average thickness of the thin films was ~300 nm.

Table 1 presents the Rhodamine 610 concentration range of the investigated thin films and of the corresponding solutions.

The Rh610 concentration (last column, Table 1) is the same for both types of solutions investigated (with and without DNA–CTMA). Previous works [14] showed that chromophore concentration of interest is around 10% with respect to the matrix. Therefore, the minimum concentration was chosen at 5%, while the highest concentration was selected at around 13%. In addition, the upper value of the Rh610 concentration in the investigated solutions is limited by the aggregation of the Rh610 molecules [29], leading to photoluminescence quenching, which is accompanied by the decrease in the light-emission quantum efficiency, a detrimental effect for photonic applications based on the light-emission process. The investigated samples have been prepared in such a way as to cover quite uniformly the entire considered range of Rh610 concentrations.

The DNA–CTMA based solutions were used also for the thin film preparation.

**Figure 1:**
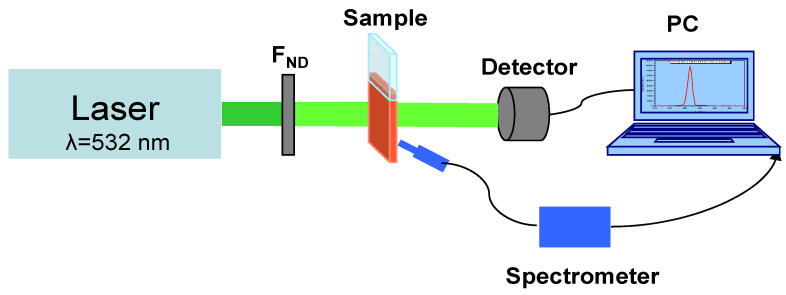
A schematic sketch of the experimental set-up used in the investigation of transmission and luminescence of DNA–CTMA–Rh610 samples.

The experimental set-up presented in Figure 1 was used to investigate the change in the transmission and of the fluorescence excited by the c.w. laser light at 532 nm wavelength in the DNA-based materials.

The wavelength of the c.w. laser beam used to excite the luminescence in the investigated samples is λ = 532 nm, which is near to the absorption maxima of the solutions of DNA–CTMA–Rh610 in butanol and Rh610 in butanol, respectively [14].

This green laser beam is the second harmonic of a Nd:YAG laser (Apel Laser SRL). It has a good temporal stability of the optical power and a near Gaussian spatial transversal profile of optical intensity with the value of the M^2^ factor very close to unity, M^2^ = 1.01.

The laser beam diameter at the sample plane is d = 0.86 mm. Calibrated absorptive neutral density filters (F_ND_, Thorlabs) are used as optical attenuators in order to adjust the power of the laser beam incident on the sample to the desired value. F_ND_ is the generic acronym for a single ND filter or a combination of ND filters, which ensure the desired optical density in each experiment. For optical alignment of each sample and of the optical detector, relative to the incident green laser beam, before starting a new transmission experiment on a different sample, the power of the laser beam is decreased at the minimum value at which the laser beam spot is still visible on the sample and on the detector. This is performed by using a single ND filter or a combination of ND filters with a higher optical density than the one used during the actual experiment. The optical alignment using a laser beam with extremely low power is necessary in order to avoid any effect photo-induced by the laser light in the sample before the start of the experiment. In order to avoid the saturation of the spectrum analyzer, the distance between the input end of the optical fiber that collects the light emitted by the sample and the sample itself is slightly tuned for each individual experiment.

The optical powers of the incident beam and of the beam transmitted through the investigated samples are measured with a computer-controlled powermeter, FieldMaxII-TOP (Coherent, Portland, OR, USA), with a power sensor OP–2–Vis, (Detector, in the setup). The power of the laser beam incident on the samples was fixed at 7 mW, to which corresponds an optical intensity of 1200 mW/cm^2^. The luminescence emission has been monitored with a spectrometer (Ocean Optics HR4000) coupled to a computer. The end of the optical fiber of the spectrometer was oriented to be near orthogonal to the direction of the excitation beam. The investigated solutions have been placed in special demountable rectangular cuvettes (Hellma, Germany) of 0.1 mm internal thickness and 26 μL internal volume with optical quality windows made from quartz. The two component parts of the cuvette (the base and the window) are fixed in a special aluminum holder, which keeps them tightened and avoids evaporation of the solution. This kind of demountable cuvette ensures both their easy filling with the investigated solutions and the facile cleaning of their interior.

## 3. Results and Discussion

**Figure 2:**
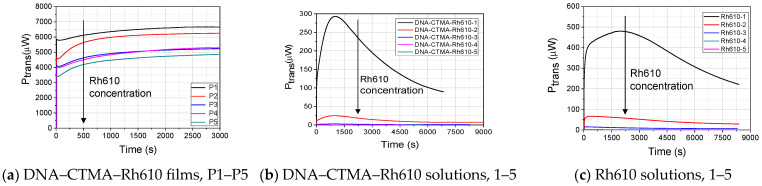
A comparison of the temporal evolution of the power, P_trans_, transmitted through P1–P5 thin films (**a**), through DNA–CTMA–Rh610 solutions used for the deposition of the films (**b**), and through Rh610 solutions with similar dye concentrations (**c**). The power incident on the samples was 7 mW (intensity of 1200 mW/cm^2^).

Using the experimental setup from Figure 1, the temporal dependence of the transmission at 532 nm, during the illumination with this light, has been studied for the thin films with an Rh610 concentration described in Table 1, for corresponding solutions DNA–CTMA–Rh610, 1–5, and for Rh610 solutions with similar dye concentrations. These temporal dependencies are shown, for comparison, in Figure 2a–c. The powers of the incident and transmitted beams have been corrected to Fresnel reflections on the involved interfaces of each considered sample.

Figure 2 reveals the major differences between the temporal evolutions of the transmission at a 532 nm wavelength in DNA–CTMA–Rh610 films, compared to the two kinds of Rh610 solutions in butanol, with and without DNA–CTMA, during their illumination with c.w. green laser light.

The obtained results are in agreement with those of You et al. [30]. Rhodamine is quite a large molecule, and therefore, there is no groove or intercalation binding with the DNA helix (as demonstrated for other small dye molecules [31]), rather it interacts with the chain of the surfactant molecule linked to DNA. This possible interaction explains the temporal behavior of the transmission as well as the differences observed between the results obtained for solutions with and without DNA–CTMA.

In the case of the investigated thin films (Figure 2a), there is an initially fast dynamic, consisting in a small decrease in transmission, which covers a time interval of several tens of seconds. After this, there is a slow component, of thousands of seconds, consisting of a continuous and asymptotic increase in the transmission of films towards constant values, larger than the initial ones.

The temporal evolution of transmission in the case of DNA–CTMA–Rh610 solutions and of Rh610 solutions, respectively (Figure 2b,c), reveals very different dynamics. Thus, there is an initial increase in transmission, on a timescale which decreases when the Rh610 concentration increases, followed by an asymptotic decrease in transmission towards constant values.

The temporal evolutions of the power transmitted through P1–P5 thin films (Figure 2a) evolve asymptotically to steady-state values faster than the temporal evolutions of the power transmitted through DNA–CTMA–Rh610 solutions used for the deposition of the films (Figure 2b), and through Rh610 solutions with similar dye concentrations (Figure 2c).

These changes in absorption for both kinds of the investigated samples, films and solutions, can be attributed to photo-induced processes experienced by the absorbing Rh610 molecules (photobleaching), accompanied by the accumulation of reaction products resulting from the photochemical decomposition of Rh610 molecules, with different absorbing properties [32,33,34]. The coexistence of these two processes with, probably, different temporal rates of change in the absorption in films and in solutions leads to the complex character of the temporal evolution of their transmission.

Photobleaching consists of fluorescent dye degradation produced by light. It is due to the fact that the dye molecules undergo irreversible modifications induced by photo-chemical reactions. As a result, both the absorption and the emission properties of the material are severely affected by the decrease in the number of dye molecules that can absorb and consequently emit light and by the appearance of non-fluorescent reaction products with different absorption properties [32,33,34].

There are several factors that have to be considered when comparing the light-induced changes in the optical absorption in the investigated films with respect to the corresponding solutions. The local heating induced by the absorption of the incident laser beam produces a temperature difference between the illuminated and the non-illuminated zones of the sample. In the investigated solutions, this temperature difference is the source of a convective heat transfer, which causes local liquid movements implying the movement of both non-photobleached Rh610 molecules and of the photochemical reaction products between the illuminated and the non-illuminated zones of the sample. These local movements induced by laser heating cannot occur in solid films.

The Gaussian transversal profile of the incident laser beam intensity induces a temperature gradient in the illuminated area due to the different radial intensity of the incident beam and also the different radial photobleaching dynamics, i.e., faster where the light intensity is higher. These effects are present in both kinds of samples, films and solutions.

The photobleaching of Rh610 molecules and the appearance of the photochemical reaction products influence the propagation of the incident laser beam along its path through the samples, as these two interrelated light-induced processes modify the light absorption and, consequently, the light intensity gradient in the depth of the samples, with different rates in films with respect to solutions.

The consideration of at least the above-mentioned processes induced by the incident laser light at a wavelength of 532 nm in the investigated samples makes the quantitative modeling of the temporal evolution of the optical absorption in the DNA-CTMA-Rh610 films and solutions and in Rh610 solutions a very difficult task.

By comparing the dynamics of light-induced changes in transmission in solutions, it is possible to see that the initial decrease in absorption is faster in Rh610 solutions than in DNA–CTMA–Rh610 solutions, suggesting a kind of protection offered by the DNA–CTMA biopolymer environment against the photobleaching of Rh610 molecules.

**Figure 3:**
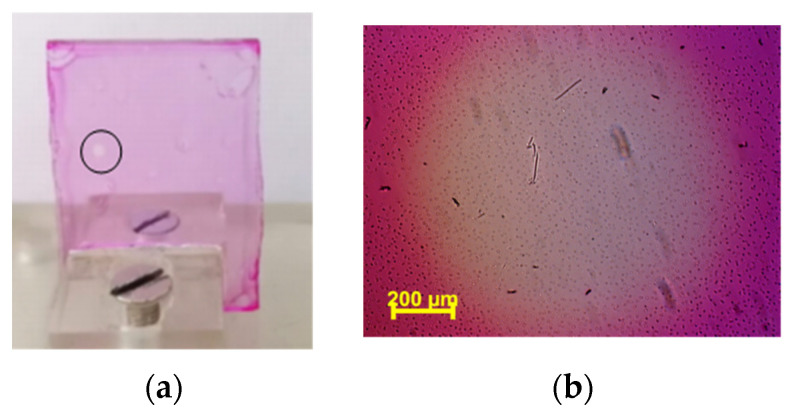
The discolored illuminated area (in the circle) (**a**) and its magnified view (**b**) of DNA–CTMA–Rh610 film (film P4), at the end of its exposure. The investigated sample (film on glass substrate) is fixed in a holder, visible in (**a**) at the bottom of the image.

In the case of films, the smallest relative change in the transmission is in the case of the lowest Rh610 concentration (5% Rh610 concentration with respect to DNA–CTMA dry mass). Visually, the DNA–CTMA–Rh610 film is discolored in the area illuminated with the light at λ = 532 nm. This discoloration is easier to be seen by the naked eye in films with a higher Rh610 concentration, i.e., higher absorption at the start of the illumination with green light. As an example, this effect is shown in Figure 3a,b, for the film P4.

The different evolution of the transmission in films and in solutions also suggests that the photochemical processes that take place during the photodegradation of the Rh610 molecules in the solid biopolymer matrix could be different from those that take place in the DNA–CTMA–Rh610 solutions in butanol.

The photochemical processes induced by c.w. laser light at λ = 532 nm affects not only the transmission through the films of the light with this wavelength but also their photoluminescence emission excited by a 532 nm laser light.

**Figure 4:**
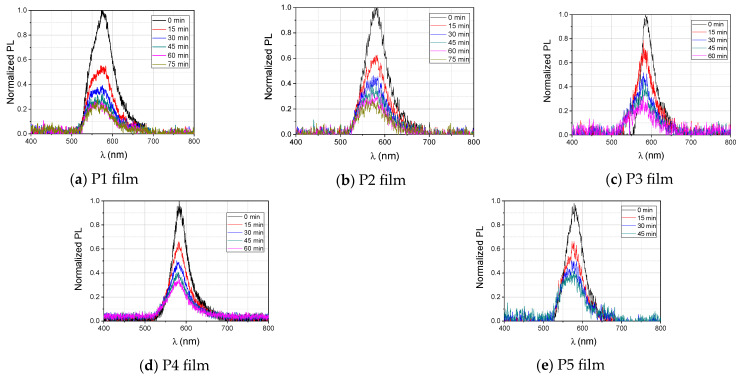
The fluorescence spectra, normalized to the peak amplitude of the initial spectrum of each sample, for films P1 (**a**), P2 (**b**), P3 (**c**), P4 (**d**), and P5 (**e**).

The temporal evolution of the fluorescence spectra of the films P1–P5 of DNA–CTMA–Rh610 during ~1 h of their excitation is shown in Figure 4. The spectra acquired at different moments, separated by time intervals of 15 min, have been normalized to the peak amplitude of the initial spectrum of each sample, acquired immediately after the start of the illumination. The normalized spectra are shown in Figure 4a–e, for the films P1–P5, respectively.

Upon excitation of Rh610 molecules with light (c.w.) at λ = 532 nm, the left side of their emission spectra is affected by the partial absorption in Rh610 of the emitted fluorescence light. This is due to the fact that the absorption and emission spectra of Rh610 are overlapping in the range of wavelengths between the excitation wavelength and approximately 575 nm [35].

**Figure 5:**
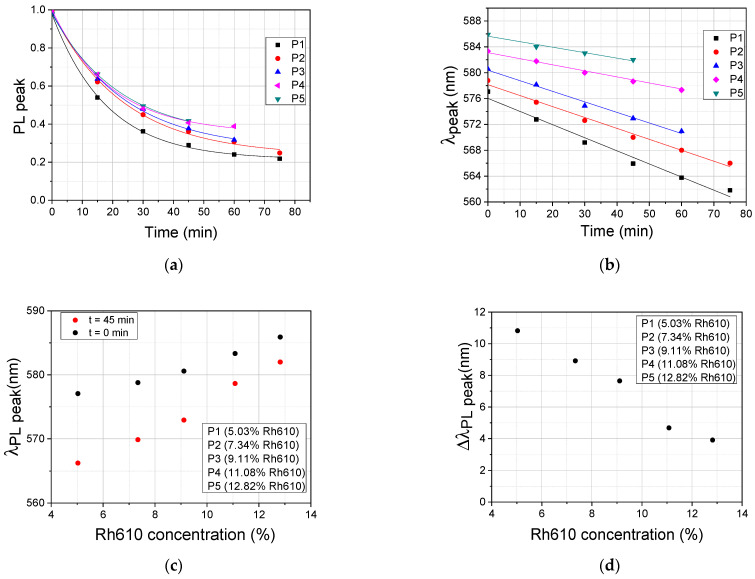
The temporal evolution of the normalized peak amplitudes (**a**), the dependence on time of the peak wavelengths (**b**), the dependence on Rh610 concentration of the wavelength of the fluorescence peaks at *t* = 0 and at 45 min after the start of photoexcitation with light at 532 nm (**c**), and the difference between these values (**d**), for the entire set of P1–P5 films.

The temporal evolution of the normalized peak amplitudes and the dependence on time of the peak wavelengths are shown together for the entire set of P1–P5 films in Figure 5a,b, respectively. The dependence on the Rh610 concentration of the wavelength of the fluorescence peaks at the initial moment of photoexcitation with light at 532 nm, *t* = 0, and at 45 min (*t* = 45 min) after the start of excitation, and the difference between these values, respectively, are shown in Figure 5c,d, and also for the entire set of P1–P5 films.

In Figure 5a, the experimental discrete points, for P1–P5 films, have been fitted with exponential decay functions, $y_{i}(t)=y_{0i}+A_{i}\cdot \exp\left(-k_{i}\cdot t\right)$, *i* = 1, …, 5, shown as continuous lines. The parameters *y*_0*i*_, *A_i_*, and *k_i_*, of the fitting functions for the experimental data from Figure 5a, corresponding to P1–P5 films, are shown in Table 2.

This kind of mathematical function, which describes the decay of the fluorescence spectra of P1–P5 films, could be a consequence of the diminishing in time of the number of fluorescing Rh610 molecules in the excited volume of the sample due to their photodegradation induced by the green light.

The kinetic of the photo-induced processes in the investigated samples is non-uniform both inside the illuminated area and along the direction of light propagation through the sample. The kinetic has a radial gradient, due to the Gaussian transversal profile of the incident laser beam, and a gradient in the depth of the sample, due to cumulative effects of linear absorption and photobleaching. Thus, the accurate quantitative analysis of the kinetic of light-induced processes in the DNA-CTMA-Rh610 films is not an easy task and requires specific modeling and calculation.

In Figure 5b, the experimental discrete points, for P1–P5 films, have been fitted with linear functions, $y_{i}(t)=a_{i}+b_{i}\cdot t$, shown as continuous lines.

The parameters *a*_i_ and *b*_i_ of the fitting functions for the experimental data from Figure 5b, corresponding to P1–P5 films, are shown in Table 3.

From the values of the slope *b_i_* (nm/min) of the fitting curve in Table 3, it is easy to see that the blue-shift in the fluorescence peaks of the investigated P1–P5 films is slower with the increase in the Rh610 concentration.

From Figure 4 and Figure 5, it can be observed that the fluorescence emission of Rh610 diminishes over time with a decreasing temporal rate followed by an asymptotic trend towards constant values after several tens of minutes.

Comparing Figure 4 and Figure 5 with Figure 2, it can be seen that the changes in the fluorescence emission continue even after the transmittances of the P1–P5 films have reached stationary states, at approximately 40–50 min after the start of their illumination with the green laser beam at a 532 nm wavelength.

The relative decrease in the normalized amplitudes of the photoluminescence peaks (the difference between the values of the normalized photoluminescence peaks at the start of the excitation and at a later time moment) corresponding to P1–P5 films is decreasing with the increase in the Rh610 concentration in films (Figure 5a).

This process is accompanied by a shift in the fluorescence peaks toward higher photon energies (lower wavelengths—blue-shift) (Figure 5b). The value of the shift decreases as the Rh610 concentration in the films increases (Figure 5c). At 45 min after the beginning of the c.w. excitation with light at 532 nm, the values of the shift in the fluorescence peak are Δλ_P1_ ≅ 10.8 nm (film P1), Δλ_P2_ ≅ 8.9 nm (film P2), Δλ_P3_ ≅ 7.6 nm (film P3), Δλ_P4_ ≅ 4.7 nm (film P4), and Δλ_P5_ ≅ 3.9 nm (film P5) (Figure 5d) for the investigated DNA–CTMA–Rh610 films.

As explained earlier, the dye has a possible interaction with the surfactant chain. If in solution, due to the mobility of the molecules, the dye is more protected, then in the case of thin films, the dye molecules are linked to the surfactant chain, and in this way, the aggregation is prevented. However, if the dye concentration is increased, the excess chromophore molecules will aggregate. As You et al. [30] explained, the phthalide group in dye molecules is easily incorporated into CTMA ligands without hindrance. After incorporation, the xanthene group, which is the fluorescing group in Rhodamine dyes, is relatively far from the DNA chain/globule.

Thus, the DNA–CTMA matrix ensures a kind of protection against the photo-induced degradation mechanisms for the Rhodamine molecules embedded in it, thus explaining the better chromophore emission and the temporal behavior in comparison with Rhodamine embedded in other synthetic polymers, e.g., PMMA [13].

This experiment revealed that the DNA–CTMA matrix has an active influence on the Rhodamine 610 emission excited by c.w. laser light at a 532 nm wavelength, in the whole range of Rh610 concentrations of the investigated films.

## 4. Conclusions

The temporal evolution of the transmission of c.w. laser radiation with λ = 532 nm through DNA–CTMA–Rh610 films with different Rh610 concentrations and of their fluorescence, when the films were excited by the same laser light, have been experimentally investigated. The comparative analysis of the transmission dynamics in films, in the solutions of DNA–CTMA–Rh610 in butanol, from which the films have been obtained, and in Rh610 solutions with the same Rh610 concentrations, reveals a major difference between films and solutions. Several factors that can contribute to different dynamics of light absorption at 532 nm in films and solutions of DNA–CTMA–Rh610 have been discussed. The different transmission dynamics also suggest that the photochemical processes, which occur in Rh610 molecules during their illumination, could be different in the solid biopolymer matrix as compared to those that occur in the DNA–CTMA–Rh610 solution in butanol and in Rh610 solution in butanol. The light-induced changes in the film’s transmission, consisting of an asymptotic increase in their transmission towards constant values, are accompanied by a reduction in their fluorescence with an asymptotic decreasing trend towards constant values. During the decrease in fluorescence emission, the spectra are blue-shifted, with the shift in the peak wavelength decreasing as the Rh610 concentration in the films increases. The relative decrease in the normalized amplitudes of the photoluminescence peaks, at the same time moments from the start of the excitation, is decreasing with the increasing Rh610 concentration in the investigated films.

The obtained results are useful for the use of DNA–CTMA–Rh610 films in applications based on fluorescence emission and lasing, and also in nonlinear photonics.

## Figures and Tables

**Table 1:** List of the investigated samples.

Film Name	Rh610 Concentration with Respect to DNA–CTMA (% Mass)	Rh610 Concentration in Solution (g/L)
P1	5.03	1.62
P2	7.34	2.42
P3	9.11	3.02
P4	11.08	3.84
P5	12.82	4.43

**Table 2:** The parameters *y*_0*i*_, *A_i_*, and *k_i_* of the fitting functions for data points from Figure 5a.

	Film	P1	P2	P3	P4	P5
Fitting Parameter						
*y*_0*i*_		0.215	0.235	0.266	0.350	0.330
*A_i_*		0.763	0.752	0.721	0.650	0.651
*k_i_* (min^−1^)		0.0555	0.0422	0.0424	0.0513	0.0451

**Table 3:** The parameters *a_i_* and *b_i_* of the fitting functions for data points from Figure 5b.

	Film	P1	P2	P3	P4	P5
Fitting Parameter						
*a_i_* (nm)		576.03	578.15	580.39	583.10	585.65
*b_i_* (nm/min)		−0.203	−0.169	−0.163	−0.094	−0.085

## Data Availability

Not applicable.
